# Supplementary material for: Automatic large-scale political bias detection of news outlets
Source: PLoS One. 2025 May 12;20(5):e0321418. doi: 10.1371/journal.pone.0321418 (PMC12068563; doi:10.1371/journal.pone.0321418)
Supplement: S1 Appendix — This appendix describes in more detail the features provided by MBFC. For each news web-domain, they provide a set of data points of interest. (PDF) [file pone.0321418.s001.pdf]

Appendix A: MBFC Features

This appendix describes in more detail the features provided by MBFC. For each news outlet, they provide a set of data points of interest:

- **Political lean** denotes whether a news outlet will tend to favor either the left or right political spectrum. This feature contains five classes in total: "left", "left center", "least biased", "right center" and "right" leaning. They compile a set of principles for determining whether a source is left or right leaning, which can be found on their website for further information [1].
- **Factuality** refers to how factual a source tends to be, whether they use credible sources, immediately correct incorrect information and have failed credible reporting fact-checks in the past. The categories span from "very low", "low", "mixed", "mostly factual", and"high" to "very high". Details of how these are evaluated are explained in their methodology.
- **Traffic** estimates are drawn from Similar Web to determine the amount of visitors each news outlet site receives, accounting for page views, print and media market viewers per month. An outlet with under 150 thousand views per month is classed as having minimal traffic, 150 thousand to 2.5 million as medium traffic and anything above is denoted as high traffic.
- **Country Press Freedom**, as measured by the World Press Freedom Index by Reporters without Borders [2]. They use each country's rank to determine their score: top ten countries receive an "excellent" score, those until top 50 a "mostly free" score, top 100 are considered to have "moderate freedom", top 160 "limited freedom" and the remainder are classed as "oppressed". There are a total of 180 countries ranked.
- **Media Type** records which types of media the news outlet in question uses. This includes many often overlapping categories that were simplified into the following classes: website, TV station, radio station, journal, magazine, news agency, news paper and organisations/foundations.
- **Credibility Rating** is a combination of some of the above mentioned features. It combines the factuality score with the traffic, political lean magnitude and press freedom scores to determine a final rating, ranging from "high credibility","medium credibility" to "low credibility".

References

1. Media Bias Fact Check. Left vs. Right Bias: How we rate the bias of media sources; 2021. Available from: `https://mediabiasfactcheck.com/left-vs-right-bias-how-we-rate-the-bias-of-media-sources/`.
2. Borders R, French N. The World Press Freedom Index 2022; 2022.
