# Supplementary material for: Automatic large-scale political bias detection of news outlets
Source: PLoS One. 2025 May 12;20(5):e0321418. doi: 10.1371/journal.pone.0321418 (PMC12068563; doi:10.1371/journal.pone.0321418)
Supplement: S2 Appendix — This appendix describes in the implementation and other details related to the data pre-processing. (PDF) [file pone.0321418.s002.pdf]

Appendix B: Details of implementation and data pre-processing

All preprocessing steps were performed using Scikit-Learn [1]. For political bias labels by MBFC, we used a total of 3,981 rated web-domains. Those with missing data, incorrect formatting or duplicates were excluded. While MBFC also rates some web-domains as being either extreme left- or right-leaning, these classes were merged with the left- and right-leaning categories due to the sparsity of instances in these classes. Joining the MBFC domains with the GDELT dataset resulted in 1,207 overlapping web-domains with more than one hundred published articles. For the PABS bias labels, the total number of web-domains with a ground truth was 3,709 when combined with web-domains present in GDELT. Web-domains that had published fewer than one hundred articles during 2022 were dropped, yielding a final total of 2,073 web-domains.

The features in the GDELT training data were normalized to range between 0 and 1 using. Country names in the MBFC features were swapped for coordinates using the Nominatim library [2]. As mentioned, there were a large amount of themes that were filtered out. The final number of themes included in our dataset 519. Given that there were multiple features associated with each theme (per theme, there is the average word, count, average polarity, etc.), the data contained a total of 6,748 features.

Given the sizeable amount of GDELT features in our experiment, some preprocessing was done to eliminate redundant features, increasing ease of fit and classification speed: columns that had a correlation score above 0.95 were removed, and recursive feature elimination was performed Using a random forest classifier with 10-fold cross-validation to find a subset of informative features [3]. The data was then split into training, testing, and validation sets, where the validation and test sets made up 15% of the total dataset size each. The sets were stratified based on political leanings.

References

1. Pedregosa F, Varoquaux G, Gramfort A, Michel V, Thirion B, Grisel O, et al. Scikit-learn: Machine learning in Python. the Journal of machine Learning research. 2011;12:2825–2830.

2. Clemens K. Geocoding with openstreetmap data. GEOProcessing 2015. 2015; p. 10.

3. Guyon I, Weston J, Barnhill S, Vapnik V. Gene Selection for Cancer Classification using Support Vector Machines. Mach Learn. 2002;46(1-3):389–422. doi:10.1023/A:1012487302797.
