# Supplementary material for: Automatic large-scale political bias detection of news outlets
Source: PLoS One. 2025 May 12;20(5):e0321418. doi: 10.1371/journal.pone.0321418 (PMC12068563; doi:10.1371/journal.pone.0321418)
Supplement: S3 Appendix — The appendix details the hyperparameters used for grid search when optimizing the various models. (PDF) [file pone.0321418.s003.pdf]

Appendix C: Grid Search Model Parameters

0.1 Support Vector Classifier

These hyperparameters of the Scikit-Learn [1] SVC model were used for the grid search:

Table 1. Support Vector Parameters

Parameter	Values
C	0.01, 0.1, 2, 4, 5, 10
gamma	0.1, 0.01, 0.001
kernel	"rbf", "poly", "linear"
degree	1, 2, 3, 4, 5, 10
break_ties	True, False
class_weight	"balanced", None

0.2 Decision Tree Classifier

These hyperparameters of the Decision Tree model were used for the grid search:

Table 2. Decision Tree Parameters

Parameter	Values
criterion	"gini", "entropy", "log_loss"
splitter	"best", "random"
max_depth	None, 3, 5, 10, 50, 100
min_samples_split	2, 5, 50, 100, 500
min_samples_leaf	1, 3, 5, 10, 50
max_features	"sqrt", "log2", None, 10, 100, 500, 1000, 2000
max_leaf_nodes	5, 10, 20, 50, None
class_weight	"balanced", None

0.3 Random Forest Classifier

These hyperparameters of the Random Forest model were used for the grid search:

Table 3. Random Forest Parameters

Parameter	Values
n_estimators	10, 50, 100
criterion	"gini", "entropy", "log_loss"
max_depth	3, 5, 10
min_samples_split	2, 5, 10
min_samples_leaf	5, 10, 20, 50
max_features	"sqrt", "log2", None, 10, 100
max_leaf_nodes	5, 10, 20, 50, None
bootstrap	True, False
warm_start	True, False

0.4 AdaBoost Classifier

These hyperparameters of the AdaBoost model were used for the grid search:

Table 4. AdaBoost Parameters

Parameter	Values			
estimators	classifier	max_depth	min_samples_leaf	class_weight
	DecisionTree	1	1	"balanced"
	DecisionTree	3	1	"balanced"
	DecisionTree	5	1	"balanced"
	RandomForest	1	1	"balanced"
	RandomForest	3	1	"balanced"
	RandomForest	5	1	"balanced"
n_estimators	5, 10, 50			
learning_rate	0.001, 0.01, 0.1, 0.2, 0.5, 1, 2			

0.5 XGBoost Classifier

The following hyperparameters of the XBGoost model were used for the grid search:

Table 5. XGBoost Parameters

Parameter	Values
n_estimators	3, 5, 10, 50
learning_rate	0.1, 0.01, 0.05

0.6 Bagging Classifier

These hyperparameters of the Bagging model were used for the grid search:

Table 6. Bagging Model Parameters

Parameter	Values
estimator	SVC, DecisonTree, RandomForest, AdaBoost, XGBoost
n_estimators	5, 10, 20, 50, 100
max_features	0.2, 0.5, 0.8, 1.0
bootstrap	True, False
bootstrap_features	True, False
warm_start	True, False

References

1. Pedregosa F, Varoquaux G, Gramfort A, Michel V, Thirion B, Grisel O, et al. Scikit-learn: Machine learning in Python. the Journal of machine Learning research. 2011;12:2825–2830.
