# Supplementary material for: Automatic large-scale political bias detection of news outlets
Source: PLoS One. 2025 May 12;20(5):e0321418. doi: 10.1371/journal.pone.0321418 (PMC12068563; doi:10.1371/journal.pone.0321418)
Supplement: S4 Appendix — This section notes the architecture of the PyTorch neural network for all experiments. (PDF) [file pone.0321418.s004.pdf]

Appendix D: Neural Network Architecture

The architecture of the PyTorch [1] neural network is as follows:

- Model: TabularNetModel
- Layers:
  - Linear layer: input features=357, output features=512
  - ReLU activation function
  - Batch normalization with 512 features
  - Dropout with probability 0.5
  - Linear layer: input features=512, output features=256
  - ReLU activation function
  - Batch normalization with 256 features
  - Dropout with probability 0.5
  - Linear layer: input features=256, output features=5
- Validation accuracy metric: MulticlassAccuracy
- Test accuracy metric: MulticlassAccuracy

For the experiment of full bias and categorical features, it was:

- Model: TabularNetModel
- Embeddings:
  - Factuality: Embedding(8, 4)
  - PressFreedom: Embedding(6, 3)
  - MediaType: Embedding(9, 5)
  - Traffic: Embedding(5, 3)
  - Credibility: Embedding(5, 3)
- Layers:
  - Linear layer: input features=377, output features=512
  - ReLU activation function
  - Batch normalization with 512 features
  - Dropout with probability 0.5
  - Linear layer: input features=512, output features=256
  - ReLU activation function
  - Batch normalization with 256 features
  - Dropout with probability 0.5
  - Linear layer: input features=256, output features=5
- Validation accuracy metric: MulticlassAccuracy
- Test accuracy metric: MulticlassAccuracy

References

1. Paszke A, Gross S, Massa F, Lerer A, Bradbury J, Chanan G, et al. PyTorch: An Imperative Style, High-Performance Deep Learning Library. In: Wallach HM, Larochelle H, Beygelzimer A, d'Alché-Buc F, Fox EB, Garnett R, editors. Advances in Neural Information Processing Systems 32: Annual Conference on Neural Information Processing Systems 2019, NeurIPS 2019, December 8-14, 2019,

Vancouver, BC, Canada; 2019. p. 8024–8035. Available from:
https://proceedings.neurips.cc/paper/2019/hash/bdbca288fee7f92f2bfa9f7012727740-Abstract.html.
