# Supplementary material for: Automatic large-scale political bias detection of news outlets
Source: PLoS One. 2025 May 12;20(5):e0321418. doi: 10.1371/journal.pone.0321418 (PMC12068563; doi:10.1371/journal.pone.0321418)
Supplement: S5 Appendix — This section notes the implementation details of the LLM baseline. (PDF) [file pone.0321418.s005.pdf]

Appendix E: Large Language Model Baseline

As a part of our analysis, we obtained experimental results using naive zero-shot LLMs. The prompt provided to the LLM is detailed below:

> You are tasked with rating various news web-domains with a label corresponding to their political bias. There are 5 possible labels you can choose from:
>
> - left (label is 0)
> - left center (label is 1)
> - least biased (label is 2)
> - right center (label is 3)
> - right (label is 4)
>
> Please assign a label to each of the news web-domains in the list provided below. Please provide the score in a valid JSON format, with no other text or responses in the output whatsoever. Example output:
> “news-domain.com": 2
>
> Here are the outlets you should label: {}

The names of the news web-domains were inserted within the brackets.
