# Supplementary material for: Automatic large-scale political bias detection of news outlets
Source: PLoS One. 2025 May 12;20(5):e0321418. doi: 10.1371/journal.pone.0321418 (PMC12068563; doi:10.1371/journal.pone.0321418)
Supplement: S6 Appendix — This section details some error analysis per political leaning label and the website traffic. (PDF) [file pone.0321418.s006.pdf]

Appendix F: Error Analysis

To better understand the strengths and limitations of the model, we examined the performance of the best performing model per political leaning label, and website traffic.

Table 1. Error rates per political bias label.

	**Error Rate**	**Instances**
Left	0.571	7
Left center	0.361	36
Least biased	0.1	60
Right center	0.333	18
Right	0.143	7

Table 2. Error rates per amount of traffic, as determined by Media Bias Fact Check.

	**Error Rate**	**Instances**
Minimal Traffic	0.179	28
Medium Traffic	0.169	65
High Traffic	0.4	35
