# Supplementary material for: Automatic large-scale political bias detection of news outlets
Source: PLoS One. 2025 May 12;20(5):e0321418. doi: 10.1371/journal.pone.0321418 (PMC12068563; doi:10.1371/journal.pone.0321418)
Supplement: S7 Appendix — Shows an excerpt from a news story as it is shown on GDELT. (PDF) [file pone.0321418.s007.pdf]

Appendix G: Example of a GDELT news item

The following is an example of a GDELT news item, prior to preprocessing and aggregation.

```
...
{V2DOCUMENTIDENTIFIER: 'https://www.grimsbytelegraph.co.uk/...',
V1THEMES: {'NEW_CONSTRUCTION'; 'CLOSURE'},
V21SOCIALIMAGEEMBEDS: 0,
V1TONE: [{
    AvgTone: 1.419,
        ...
    Polarity: 3.943,
    ActRefDens: 27.129,
    SelfRefDens: 3.312,
    WordCount: 587}]
}
```

Listing 1. GDELT database example prior to preprocessing.
