# Supplementary material for: Automatic large-scale political bias detection of news outlets
Source: PLoS One. 2025 May 12;20(5):e0321418. doi: 10.1371/journal.pone.0321418 (PMC12068563; doi:10.1371/journal.pone.0321418)
Supplement: Attachment — Submitted filename: Response to Reviewers.pdf [file pone.0321418.s008.pdf]

Response to Reviewers

This document details our responses per point raised by reviewers. We want to firstly thank reviewers for their insightful comments and suggestions, and we have attempted to address each point in the revised version of the paper.

We would firstly like to thank the editor for their assistance on this submission, and have made modifications relating to the feedback. Namely, we have made a minimal dataset available necessary for future replication purposes at a public repository (https://github.com/rtronnback/automatic_news_monitoring_with_GDELT). Similarly, we have verified that all pictures pass through the PACE tool and uploaded the PACE generated figures. We also ensured that figures and tables were all included immediately after they were mentioned, instead of LaTeX's default placements, and corrected file names.

We thank **Reviewer 1** for their useful feedback, we appreciated the reviewer's awareness of the broader context and the considerations for making our approach more applicable for wider use, particularly regarding systems that do not follow the Left-Right political divide. We think the paper has greatly improved thanks to their suggestions. In response to the comments, we have added guidance on the use of our approach in a cross-country setting (and refer to previous work that explores criticisms of the left-right political divide, see **lines 499-500**). Namely, users should be aware that the left-right political divide our project is based on uses the bias labels made by from Media Bias Fact Check. They provide an overview of contrasting left-right positions that is informative for deciding whether this political scale will be fully applicable to a country of interest. Given the persistence of the left-right divide, as per (Le Gall & Berton, 2013), it can be assumed that these ground truth labels are helpful in the majority of, at the very least, Western countries. We advise people interested in other divides to use their own GT labels, as our approach will still be applicable (**lines 508-519**). As we make our code fully available on GitHub, our method is easily accessible for alternative approaches.

We have, as suggested, added a specification about unexpected biases (**lines 22-23**), and the benefits of cost-effective and recurrent analyses (**lines 405-408; 472-477**). Regarding the relevance of what is covered versus not covered to determine bias, we have clarified this in the Methods section (**lines 213-222**).

We have added nuance related to the fact that no benchmarks are perfect, and suggested alternative approaches (**lines 245-249; 508-519**). We have also highlighted the finding that models perform better on human-made labels, and that this might be due to computational labels adding noise that complicate downstream analyses (**lines 457-461**).

We have elaborated on results in Figures 5 and 6 (now split into individual figures) and added a new example of a misclassified result. We have also fixed references to Figure 6.

Regarding the point of how this approach can be scaled up, we have highlighted the benefits of using GDELT, as this allows for global application of our approach (**lines 472-477; 497-519**).

We thank **Reviewer 2** for their insightful suggestions regarding how to strengthen our paper, especially for pointing out some aspects which were not sufficiently clear in the original version. In response to the comments, we'd like to reaffirm that our paper does examine skewness in the chosen GDELT features (since the systematic presence of absence of features are linked to a political bias label by the ML models), and the revisions have hopefully clarified this.

Additionally, we have added a definition of bias from the literature that better reflects the broad and nuanced nature of the subject, while improving clarity for readers (**lines 61-63**). We have added the recommended papers regarding LLM stochasticity, and are particularly appreciative of the recommendation of the work by Makhortykh et al. (2024).

Regarding the generalizability of our approach beyond journalistic materials, we have specified in the paper that our current approach extends to what is covered by GDELT. As GDELT is a uniquely detailed source, any project looking to extend a similar analysis beyond news outlets would have to first compile a dataset on the other topic in question using a similar structure as GDELT. However, GDELT is quite extensive – there are trillions of datapoints in their database, and a single year totals 2.5TB of data before aggregation. Therefore, we hope that it will cover most use cases that involve any online content (**see lines 189-200; 494-519**).

We have specified when a paper we mention has also used GDELT or Media Bias Fact Check, and added examples of existing work that relies on it to demonstrate that they are reliable tools (**lines 190-192; 249-250; 491-492**). We have also mentioned some common SHAP alternative methods used for model explainability (**lines 287-289).**

We thank **Reviewer 3** for the clarity, depth, and constructiveness of their comments. The feedback has been greatly helpful during our revision of the paper. In response to the comments, regarding point (a) and the selection bias resulting from using GDELT and a pandemic-year, we have elaborated that GDELT is currently the single largest database covering news on a global scale, and which we are confident mitigates selection bias, even despite the effects of the COVID-19 outbreak (**lines 202-205**).

For point (b), we have expanded upon the literature section as suggested, and would like to thank the reviewer for the recommendations of related papers. We have added the relevant papers (**lines 111-115; 127-141; 145-158)** as suggested by reviewer 3. Particularly the work by Ye and Skiena (2019) was examined and compared to our work, and some interesting differences were elaborated upon. It was however ultimately not added as a baseline. Despite the original paper using a sentiment score between -1 and 1, the published database transforms this to a bias rank, thus removing distinction of the left-right divide that out approach relies on. Due to this, we cannot compare the two approaches, since it provides only an estimate of the amplitude of bias and not its direction, as our work does. Therefore, comparison would necessitate a fundamental change for our approach that is not compatible with the current focus on a left-right political divide. Furthermore, few domains in our test set were to be found in the database, making comparison difficult. As mentioned above, however, future projects can use our code for such alternative ground truth labels, should that be the point of focus.

For point (c), we have added examples of errors with analysis as a way of interpreting the model's behaviour, particularly for the traffic of the domain, and the classification label (**see Appendix F**). We have also added a demonstrative example of using SHAP to examine misclassifications (**see Figure 10**).

As per point (d) in suggested edits, we have added an example of the GDELT data before it is aggregated to **Appendix G**. Furthermore, the Results section now includes **Table 3** which shows an overview of domains, their true labels, and the classifications by our best model.

Regarding point (e) and the addition of a zero-shot LLM experiment, we have taken the reviewer's suggestions, refined the text (**lines 142-165**), and performed a baseline using Llama 3.1 and GPT4-io mini (**see Table 2, and Appendix E**). Given that the GDELT aggregate data used

to train the other models is purely numerical, we opted for a naïve zero-shot baseline using the domain name, thereby relying on the LLM's latent representations of the domains. We have, during other projects, experimented with using LLMs in this way with more elaborate prompts, but have to date only achieved middling results at best.

As per point (f), the code used for this analysis, as well as the training, validation and testing data, is available on Github (see footnote on page **8**).

We once again wish to heartily thank the editors and reviewers for their valuable feedback that has allowed us to noticeably improve on the paper.

References:

Le Gall C, Berton R. Left-Right vs. traditional and new cleavages: testing durability of an old political category. In: Left and right : the great dichotomy revisted. Cambridge Scholars Publishing; 2013.

Makhortykh M, Sydorova M, Baghumyan A, Vziatysheva V, Kuznetsova E. Stochastic lies: How LLM-powered chatbots deal with Russian disinformation about the war in Ukraine. Harvard Kennedy School Misinformation Review;doi:10.37016/mr-2020-154.

Ye J, Skiena S. MediaRank: Computational Ranking of Online News Sources. In: Teredesai A, Kumar V, Li Y, Rosales R, Terzi E, Karypis G, editors. Proceedings of the 25th ACM SIGKDD International Conference on Knowledge Discovery & Data Mining, KDD 2019, Anchorage, AK, USA, August 4-8, 2019. ACM; 2019. p.2469–2477. Available from: ttps://doi.org/10.1145/3292500.3330709.
